# Inconsistent radiotherapy effects between primary tumors and axillary lymph nodes

**DOI:** 10.20892/j.issn.2095-3941.2022.0590

**Published:** 2022-12-12

**Authors:** Zhao Bi, Pengfei Qiu, Zhaopeng Zhang, Yongsheng Wang

**Affiliations:** 1 Breast Cancer Center, Shandong Cancer Hospital and Institute, Shandong First Medical University and Shandong Academy of Medical Sciences, Jinan 250000, China

## Negative surgical margins are important for patients treated with breast-conserving therapy (BCT)

BCT has been a standard practice in breast cancer treatment for more than 2 decades. For patients who receive BCT, a positive surgical margin is defined by the presence of ink at the surfaces of surgical specimens, in either the invasive tumor cells or breast ductal carcinoma *in situ*, thus implying potentially incomplete resection, which is associated with a significantly elevated risk of ipsilateral breast tumor recurrence (IBTR)^1,2^. A meta-analysis with a median follow-up of 6.6 years has reported an odds ratio (OR) of 1.96 [95% confidence interval (CI): 1.72–2.24] for IBTR in patients with positive rather than negative surgical margins (no ink on the tumors)^3^.

The European Organization for Research and Treatment of Cancer trial has demonstrated that whole-breast irradiation (WBI) and an additional boost dose of 16 Gy in the tumor bed after complete tumor resection significantly decreases the rate of IBTR^4^. The overall cumulative incidence of IBTR with or without a boost in the tumor bed at 10 years was 6.2% (95% CI: 4.9%–7.5%) and 10.2% (95% CI: 8.7%–11.8%), respectively (*P* < 0.0001). In a small subset of 251 patients with positive surgical margins and those who received a boost, the cumulative incidence of IBTR at 10 years was 17.5% (95% CI: 10.4%–24.6%) with 10 Gy and 10.8% (95% CI: 5.2%–16.4%) with 26 Gy^5^. These data suggest that although a boost decreases IBTR when the margins are microscopically positive, the absolute benefit is insufficient to decrease the rate of IBTR below that in patients with negative surgical margins and the use of a boost.

Meta-analyses of surgical margins and other retrospective studies have shown that patients with positive surgical margins who have favorable biological characteristics, such as those with tumors that are strongly estrogen receptor (ER) positive, still remain at higher risk of IBTR than similar patients with negative surgical margins despite good biological characteristics^3^. A meta-analysis of 19 studies including detailed information on ER status has reported a significantly elevated adjusted OR for IBTR among patients with ER-positive tumors with positive rather than negative margins, at 2.66 (*P* < 0.001).

Therefore, surgical margin status remains a major factor for IBTR, and negative surgical margins significantly decrease the risk of IBTR.

## Patients with involvement of 1 or 2 sentinel lymph nodes (SLNs) can safely avoid axillary lymph node dissection (ALND)

Historically, ALND was the standard for management of patients with SLN metastasis, to fully understand the lymph node metastasis status and increase local control^6^. However, several randomized, controlled trials, such as ACSOG Z0011, AMAROS, and OTOASOR, have strikingly demonstrated no difference in axillary regional recurrence or overall survival between patients with early breast cancer with limited SLN involvement (1 or 2 positive SLNs) with or without ALND^7–9^. In these trials, patients with limited SLN involvement were randomly assigned to receive either ALND or no further axillary dissection. In the group receiving no further axillary dissection, radiotherapy plus adjuvant systemic therapy was used with a median follow-up of 10 years, and the rates of axillary regional recurrence were 1.1%, 1.8%, and 1.7% (8 years) in the ACSOG Z0011, AMAROS, and OTOASOR trials, respectively^7–9^. In summary, these trials have shown that omission of ALND, followed by radiotherapy and adjuvant systemic therapy, is safe and is not associated with any difference in regional recurrence in patients with early breast cancer and limited SLN involvement^10^. Axillary recurrence is low even in patients undergoing axillary de-escalation surgery, thus suggesting that tumor biology, adjuvant systemic treatment, and radiotherapy may potentially have crucial roles^6^.

## Thirty percent of patients with 1 or 2 positive SLNs may experience non-SLN metastasis

**Figure 1:**
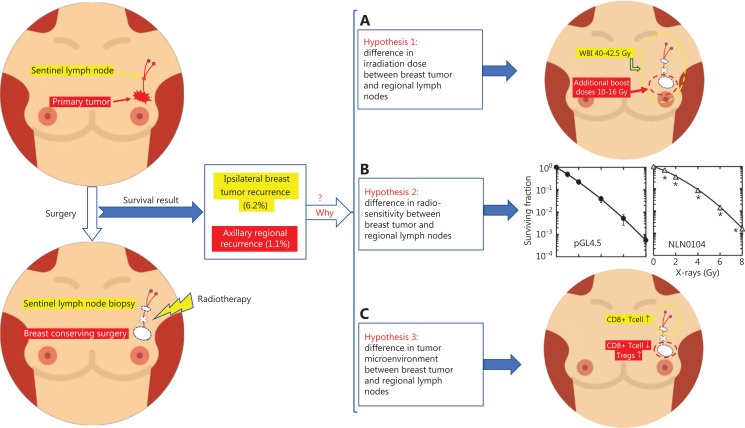
Hypotheses potentially explaining the difference in recurrence rates between the primary tumor and axilla regional nodes. (A) The first hypothesis is a difference in irradiation dose between breast tumors and regional lymph nodes. (B) NLN0104 cells (established a cell line metastatic to the lymph nodes) are more resistant to X-ray-induced clonogenic inactivation *in vitro* than primary tumor cells (pGL4.5). (C) In the axilla region, CD8+ T cells are up-regulated, whereas in the primary tumor region, CD8+ T cells are down-regulated, and Tregs are diminished.

In patients with negative surgical margins (no ink on tumors) and 1 or 2 positive SLNs without ALND who received BCT, the residual tumor burden of the axilla region was higher than that of the primary tumor. However, the incidence of axilla regional recurrence was much lower than that of IBTR (1.1% *vs*. 6.2%). Several hypotheses have been proposed to explain this phenomenon (**Figure 1**), as follows:

### Hypothesis 1: The difference in irradiation dose between breast tumor and axilla regional nodes

The first hypothesis is that a difference in irradiation dose between breast tumor and regional lymph nodes (**Figure 1A**) might explain why the axillary regional recurrence rate was far lower than the IBTR rate. In the Z0011 trial, patients who underwent SLNB were randomly (1:1) assigned to ALND or axillary radiotherapy (SLNB) groups. In the SLNB group, patients received high-tangential WBI^7^. The National Comprehensive Cancer Network guidelines suggest that, for patients receiving WBI, the whole breast should receive a hypo-fractionated dose of 40–42.5 Gy in 15 or 16 fractions^11^. With high-tangential WBI, the axilla regional nodes also received a similar dose. Simultaneously, a boost to the tumor bed was recommended in patients at higher risk of recurrence. Additional boost doses were 10–16 Gy in 4–8 fractions. For patients who received BCT, the boost dose for the tumor bed was higher than that for regional lymph nodes. However, the incidence of breast tumor recurrence was also much higher than that of regional lymph node recurrence. Therefore, the difference in irradiation dose may not be sufficient to explain the inconsistency in the recurrence rate between the axilla and ipsilateral breast.

### Hypothesis 2: The difference in radiosensitivity between breast tumor and regional lymph nodes

The second hypothesis suggests a potential difference in radiosensitivity between the breast tumor and regional lymph nodes. Radiotherapy is used for primary tumor metastasis, with a dose determined according to the characteristics of the primary cancer, but not metastatic cancer, on the basis of clinical experience. The effects of radiotherapy might vary because of differences in radiosensitivity. Several studies to date have examined the ionizing radiosensitivity of primary and metastatic cancers. Rantanen et al.^12^ have compared clonogenic survival after X-ray irradiation of UT-EC-2A (established from a patient with endometrial adenocarcinoma) and UT-EC-2B (established from the same patient, but from the left supraclavicular fossa metastasis 17 months later). Huerta et al.^13^ have compared SW480 (established from a patient with primary colon adenocarcinoma) with SW620 (established from the same patient, but from the lymph node metastasis 6 months later). The results of these 2 studies have shown that UT-EC-2B and SW620 are more resistant to γ-induced apoptosis than primary tumor cells. Similarly, Hara et al.^14^ have established metastatic cell lines from the MB-231 cell line and characterized their radiosensitivity. The results indicated that NLN0104 cells (which established a cell line metastatic to the lymph nodes) were more chemotactic, invasive, and resistant to X-ray-induced clonogenic inactivation than pGL4.5 cells (primary cells), a finding partly attributable to the resistance to radiation-induced apoptosis (**Figure 1B**). However, the incidence of metastatic lymph node recurrence was much lower than that of primary tumor recurrence. Therefore, the difference in radiosensitivity might also be insufficient to explain the inconsistency in recurrence rate between the axilla and ipsilateral breast.

### Hypothesis 3: Differences in the tumor microenvironment (TME) between breast tumors and regional lymph nodes

Cell death induced by surgery can result in the release of cytokines. Simultaneously, innate immune cells can trigger inflammatory pathways, antibacterial immunity, and adaptive immune cell responses^15^. Post-surgical trauma may lead to T-cell dysfunction, manifested as inability to recognize antigens, decreased expression of T-cell membrane receptors, and diminished interferon-γ proliferation and production^16,17^. Surgical stress may also decrease the levels of CD8+ T cells that produce cytokines after exposure to tumor-associated antigens^18^. The induction of tumor equilibrium is one prevailing view. Equilibrium is a state in which tumor proliferation is balanced by cell death, and both angiogenic and immunological mechanisms have been demonstrated to mediate tumor equilibrium^19^. Therefore, the induction of equilibrium can represent a temporarily stable yet transitional disease state during which tumor progression is halted, but tumors eventually escape and relapse locally at variable intervals. CD8+ T cells have an essential role in achieving and maintaining progression-free disease, because CD8+ T cell depletion can lead to rapid tumor growth.

Regulatory T cells (Tregs) participate in an important mechanism regulating immune system homeostasis and the immune tolerance of the body, and play a crucial role in the regulation of tumor immunity. Tregs suppress anticancer immunity, thereby hindering protective immunosurveillance of tumors and effective antitumor immune responses in tumor-bearing hosts. Simultaneously, Tregs inhibit the activation and differentiation of CD4+ helper and CD8+ cytotoxic T cells, thus inducing reactivity against autologous and tumor-expressed antigens^20,21^. In the TME, Tregs have strong immunosuppressive function, and they are induced from traditional T cells. At the same time, Tregs can inhibit antitumor immunity, and promote the occurrence and development of tumors. Tregs also suppress immune effector cell function through a variety of mechanisms and are key factors in tumor immune escape. The specific elimination of Tregs *in vivo* effectively stimulates the antitumor immune response in patients^19^.

The postoperative amplification of Tregs upregulates caspase-3 and promotes immunosuppression and apoptosis^22^. However, for patients who receive SLNB without ALND, local surgery of the axilla does not completely destroy the structure of the lymph nodes and lymphatic vessels. Local stress might stimulate T cell subsets in the axilla region and lead to the up-regulation of CD8+ T cells, thereby enhancing antitumor ability (**Figure 1C**). Simultaneously, the TME around the axilla region might also be affected, thus decreasing the inhibition of CD8+ T cells by Tregs in the TME. Therefore, the inhibition of cancer cells in the axilla can be enhanced.

In conclusion, for patients with negative surgical margins and 1 or 2 positive SLNs without ALND who received BCT, the residual axilla tumor burden is higher than that of the primary tumor, whereas the recurrence rate in the axilla is much lower than that in the primary tumor. Findings suggest that the difference in the TME between the axilla and primary tumor might be the main reason underlying this phenomenon.
